# mHealth series: New ideas for mHealth data collection implementation in low– and middle–income countries

**DOI:** 10.7189/jogh.03.020101

**Published:** 2013-12

**Authors:** Michelle Helena van Velthoven, Josip Car, Yanfeng Zhang, Ana Marušić

**Affiliations:** 1 Global eHealth Unit, Department of Primary Care and Public Health, Imperial College London, London, UK; 2 Department of Integrated Early Childhood Development, Capital Institute of Paediatrics, Beijing, China; 3 Croatian Centre for Global Health, University of Split School of Medicine, Split, Croatia; 4 Department of Research in Biomedicine and Health, University of Split School of Medicine, Split, Croatia

## Abstract

The use of mobile devices in healthcare, or mHealth, has the potential to play an important role in low– and middle–income countries in a wide range of areas. A particular area with great potential to improve global health is using mHealth for data collection. We propose three ideas: (i) to validate and conduct household surveys, (ii) to monitor large–scale programs, and (iii) to measure the global burden of disease. We need to know more about mHealth interventions and their validity to maximise their potential.

The use of mobile devices in health care, or mHealth, has the potential to play an important role in low– and middle–income countries. The widespread use of mobile devices and almost universal coverage of the world’s population by a network signal has given mHealth a great opportunity to improve global health. There is a very significant interest in using mHealth as an additional strategy for strengthening health systems and achieving the Millennium Development Goals in low– and middle–income countries. However, mHealth has not reached its promised potential [1].

While there is a large amount of literature in the mHealth field in a wide range of areas, many mHealth projects have been small–scale pilots and very few have been thoroughly evaluated. The results of mHealth studies have been mixed and there is insufficient evidence of mHealth impact on health indicators [1,2]. The difficulties in defining and evaluating mHealth reflect on broader challenges in interpreting the complex interactions between technology, health care service designs and people. An additional problem for mHealth in low– and middle–income countries is that more mHealth efforts and evaluations have taken place in high–income countries [1].

More efforts should focus on mHealth data collection, because mHealth has great potential to improve the availability and quality of health data [3]. Accurate data are needed to evaluate health, assess effectiveness of interventions, monitor trends, inform health policy, and set priorities. However, data are often lacking and of insufficient quality, because data collection faces several challenges. Some of these challenges are inherently caused by the current data collection methods, such as face–to–face and pen–and–paper methods. Current data collection is limited by resource constraints, both in terms of personnel and financial costs. There is a short supply of trained and qualified interviewers and health workers, and data collection is often perceived as time–consuming, complex, inflexible and not useful. As a result, data are frequently fragmented, inaccessible, incomplete, and prone to error [4].

To address the problem of resource constraints, mHealth could offer a scalable solution needing minimal personnel, time, and financial resources to collect data. mHealth data collection may be more convenient for data collectors and those who provide information. Moreover, mHealth opens up new possibilities to validate and collect data. We propose three ideas where mHealth could be implemented for global health: (i) to validate and conduct household surveys, (ii) to monitor large–scale health programs, and (iii) to measure the global burden of disease.

First, large–scale household surveys are needed to provide accurate estimates of health and to measure coverage of health interventions. Population–based data that are derived from these surveys will continue to provide information in the foreseeable future in low– and middle–income countries, even when health information systems improve [5]. Surveys are needed to validate data obtained through other sources; for example information on care–seeking of caregivers for their child’s health, which is particularly limited in terms of quality and quantity [6].

Currently, some health indicators may not provide fully accurate results. Efforts to learn more about measurement using innovative designs to asses accuracy would be welcomed [5]. mHealth could evaluate and improve accuracy of reporting [7]. Mobile devices could be used to show caregivers videos on dangers signs for which care needs to be sought and this can also serve as a health education intervention to improve care–seeking behaviour. In addition, mobile phones could validate reports on care–seeking by monitoring caregivers’ health care attendance via a mobile phone and comparing this data with information about caregivers’ care–seeking behaviour collected by household surveys.

Mobile phone text messaging could be a cheaper and quicker method to conduct surveys, because no households have to be visited. In this issue of the *Journal of Global Health*, we explored how maternal, newborn and child health coverage can be measured by text messaging in China. Text messaging data collection can eliminate interviewer bias, and could increase sample size and representativeness by including hard–to–reach populations [8].

Second, effectively monitoring and evaluating large–scale health interventions is essential for planning and management of programs and to achieve high coverage of key health interventions. There are a number of large–scale international efforts that aim to improve health in low– and middle–income countries, but monitoring these efforts is often challenging. Current collection of data is time–consuming, slow and often provides out–of–date information. Continuous data collection would be particularly beneficial for evaluating interventions at scale [9]. mHealth could facilitate evaluation of these efforts by allowing data to be collected in real time [10].

Third, there is a need for global, national and regional information about the burden of disease. Especially regional data are needed, because there is substantial geographical variation in the causes of deaths, and appropriate interventions have to be tailored to a specific context to be effective. However, there are large gaps in the burden of disease, particularly in low– and middle–income countries where most deaths occur. Moreover, the local relevance of much health information is questionable [3]. The ubiquity of mobile phones allows easy scale–up of data collection and its use in different settings. mHealth interventions could collect data for different data sources that are used for global burden of disease measurements.

However, before implementing these mHealth applications, we need to know more about the evaluation of mHealth interventions and their validity to maximise their potential [2]. More field sites are needed that rigorously evaluate and validate mHealth for implementation of effective mHealth interventions. Therefore, we set up a collaboration between researchers in China and in the UK to evaluate mHealth in a field site in rural China. In this issue of the *Journal of Global Health*, we introduced the aims and objectives of our mHealth project, the field site, and the detailed methods of two studies that we conducted [11].

In the first study, we explored factors influencing sample size calculations for mHealth studies [12]. Realistic sample size calculations are essential to conduct mHealth–based studies, but we had very little information available that could be used to inform our sample size calculation. Participants can be lost during different steps in mHealth studies, including collection of names of potential participants and their mobile phone numbers, recruitment, and data collection and follow–up [8]. There are several factors influencing whether people are lost during each step; for example the proportion of people that own and are able to use a mobile phone, how they are recruited, their response rate, and willingness to participate over time. We used mixed methods to explore factors in the different steps. This work can help future mHealth studies with estimating their sample sizes.

In the second study, we determined the validity of mHealth text messaging data collection. The effects on data quality of this mode of data collection are unknown and the validity of a new data collection mode needs to be established. We conducted a cluster randomised cross–over study and included a large sample of participants. We compared a text messaging survey vs a face–to–face survey and assessed a range of outcomes: data equivalence, the amount of information in responses, reasons for different responses, the response rate, characteristics of non–responders, and error rate. This work can help future mHealth studies with developing their mHealth interventions.

The potential that the widespread use of mobile phones offers to health care should go beyond exploration. mHealth efforts should focus on effective use of validated mHealth interventions to improve health in low– and middle–income countries. The three areas in which we think that mHealth could play a significant role for global health are to validate and conduct household surveys, to monitor large–scale programs, and to measure the global burden of disease. We set up a mHealth project in rural China to rigorously evaluate and validate an mHealth intervention and contribute to the evidence base of mHealth for global health. In the next years, we hope that more efforts will evaluate mHealth sufficiently, so that it can be successfully implemented.
